# Motives matter: A smartphone EMA study on the association between motives to exercise, exercise engagement and mood

**DOI:** 10.1111/bjhp.70111

**Published:** 2026-10-05

**Authors:** Leonardo Pimpini, Ella Ferrand, Codrin Mironiuc, Jan Schepers, Anne Roefs

**Affiliations:** ^1^ Department of Clinical Psychological Science, Faculty of Psychology and Neuroscience Maastricht University Maastricht the Netherlands; ^2^ Department of Methodology and Statistics, Faculty of Psychology and Neuroscience Maastricht University Maastricht the Netherlands

**Keywords:** appearance, ecological momentary assessment, exercise, functionality, motives, networks

## Abstract

**Objective:**

Regular exercise is vital for preventing cardiovascular disease, maintaining a healthy body weight and supporting mental well‐being, yet most people exercise insufficiently often due to motivational barriers.

**Design:**

This study examined two types of exercise motive—functional (divided into health/fitness and enjoyment) and appearance—and their interplay with mood and exercise engagement in daily life. Two hypotheses were tested: (H1) functional motives, compared to appearance motive, predict greater exercise engagement, and (H2) higher (more positive) mood, in interaction with exercise motives, predicts greater exercise engagement.

**Method:**

Ninety‐eight healthy BMI women completed a 2‐week smartphone Ecological Momentary Assessment (EMA) protocol reporting exercise motive (baseline), exercise engagement (frequency, intensity, duration) and mood several times/day.

**Results:**

Partially supporting H1, enjoyment motive predicted greater exercise frequency and duration, whereas—contrary to H1— health/fitness motive predicted shorter duration and appearance was unrelated to engagement. H2 was not supported: mood did not predict engagement and functional motives did not strengthen the mood–engagement link. Contrary to H2, the appearance motive strengthened the (same‐moment) within‐person association between mood and exercise intensity, such that women higher in appearance motive showed a stronger positive mood–intensity link. Multilevel temporal networks revealed that higher (more positive) mood at one assessment weakly predicted greater exercise intensity and duration at the next assessment.

**Conclusions:**

Together, enjoyment emerges as central to exercise engagement, while positive mood temporally precedes exercise, informing future lifestyle interventions. Further research on the temporal dynamics linking mood and exercise is warranted.

**Statement of Contribution**

**What is already known?**

- Functional exercise motives, compared to appearance motives, aid exercise engagement and well‐being.
- Self‐determination theory links autonomous (functional) motives to sustained engagement.
- Evidence is mostly cross‐sectional, missing daily–life and temporal mood–exercise dynamics.

**What does this study add?**

- Intensive 2‐week assessment of exercise and mood via smartphone ecological momentary assessment.
- Combines multilevel models with network analysis across momentary and lagged levels.
- Unlike cross‐sectional designs, this study informs on the temporal link between mood and exercise.

## INTRODUCTION

### Exercise benefits and the engagement gap

Physical activity and exercise play a pivotal role in the prevention and treatment of both non‐communicable diseases and related risk factors such as overweight and obesity (Anderson & Durstine, 2019; Box et al., 2019; Guthold et al., 2018; Vermote et al., 2018). Moreover, the benefits of physical activity and exercise are not only confined to the sphere of physical health (e.g. weight management) but extend to mental health as well with positive effects on mood and mental state (Hearing et al., 2016; Lanoye et al., 2019; Liao et al., 2017; Strasser & Fuchs, 2015; Tylka & Homan, 2015; Uchida et al., 2012; Vartanian et al., 2012). Therefore, physical activity and exercise should be an integral part of people's daily lives, preferably across the entire lifespan (Dunton et al., 2018). Notably, while physical activity includes everyday activities like walking, taking the stairs or gardening (Box et al., 2019; Schultchen et al., 2019), exercise refers to repetitive, intentional movement aimed at improving or maintaining people's fitness (Box et al., 2019; Faulkner et al., 2021; Trott et al., 2020). However, despite the numerous and renowned benefits, most people fail to engage in physical activity and/or exercise on a regular basis (Box et al., 2019; Brand & Ekkekakis, 2018; Huellemann et al., 2021; Zervou et al., 2017). Therefore, the goal of this study is to examine the link between exercise motives, exercise engagement and mood in daily life among women with a healthy weight.

The World Health Organization (WHO) guidelines recommend at least 150 min of moderate aerobic physical activity per week or 75 min of vigorous physical activity. In addition, 2 days of muscle‐strengthening exercise per week would lead to optimal health benefits (Bull et al., 2020; Guthold et al., 2018). However, these guidelines are not met by the majority of the Western world's population (Degroote et al., 2020; Ndupu et al., 2023). More specifically, 40% of the adult population in Europe (Marques et al., 2015; Panão & Carraça, 2020) and 80% in the United States (Liao et al., 2017) do not meet these guidelines. Moreover, women engage in substantially less physical activity than men despite deriving greater cardiovascular benefits from it (Ji et al., 2024; Ley, 2020; Mielke et al., 2018). This disparity is driven by structural and individual‐level barriers, including caregiving responsibilities (Brown & Trost, 2003; White et al., 2005) and limited time and access to exercise environments. All in all, the reasons why those guidelines are not met are multiple and complex. Arguably, the current *obesogenic* environment (Kadouh & Acosta, 2017; Meldrum et al., 2017; Nicolaidis, 2019) plays a role, that is, an environment characterized by cheap and easily accessible high‐calorie palatable foods in addition to stress and a sedentary lifestyle (Ndupu et al., 2023; Schultchen et al., 2019; Teixeira et al., 2012).

### Exercise motives and exercise engagement (H1)

Another important aspect to consider is the *motive*, that is, the reason for which people exercise (Fuller‐Tyszkiewicz et al., 2018; Ley, 2020; Zervou et al., 2017). Motives are typically divided into three categories: enjoyment, health‐related and appearance‐related (i.e. body image, attractiveness, weight control), whereby the appearance motive has been associated with negative psychological outcomes (Box et al., 2019; Caglar et al., 2009; Craft et al., 2014; Fuller‐Tyszkiewicz et al., 2018; Huellemann et al., 2021; Vartanian et al., 2012). Notably, previous research showed that exercising for appearance reasons (e.g. weight control, toning, attractiveness) has been associated with greater body dissatisfaction (Fuller‐Tyszkiewicz et al., 2018; LePage & Crowther, 2010; Prichard & Tiggemann, 2008), signs of restrictive and dysregulated eating behaviours (Vartanian et al., 2012) and negative affect (LePage & Crowther, 2010). In contrast, exercising for functional reasons, such as health/fitness and/or enjoyment (Brunet & Sabiston, 2011; Tylka & Homan, 2015), was linked to greater body appreciation (Alleva & Tylka, 2021; Tylka & Homan, 2015), and lower stress levels (Tylka & Homan, 2015). Finally, people exercising for functional reasons seem to exercise more frequently compared to individuals focusing on appearance improvement (Box et al., 2019; O'Hara et al., 2014; Vartanian et al., 2012). Hence, the extent to which physical and psychological benefits of exercise are attained may depend on the underlying *motive to* exercise (Craft et al., 2014; Huellemann et al., 2021; Tylka & Homan, 2015).

Appearance motives are more commonly endorsed among women compared to men (Craft et al., 2014; Huellemann et al., 2021; LePage & Crowther, 2010; MacIntyre et al., 2020). Women are regularly confronted with cultural, societal and interpersonal pressures to conform to idealized body types (Homan & Tylka, 2014; Magnus et al., 2010; O'Hara et al., 2014) whereby a lean and toned shape is constantly emphasized (Craft et al., 2014; Raggatt et al., 2018). Thus, exercise is often viewed as a strategy for women to align to appearance‐related ideals (Huellemann et al., 2021). Although this can initially increase their exercise engagement, it will likely undermine adherence over time (Magnus et al., 2010). Importantly, while appearance motives are also common among men, women consistently endorse weight, shape and toning exercise more strongly than men (Craft et al., 2014; Vartanian et al., 2012). Growing evidence demonstrates that appearance‐based motives to exercise are more robustly associated with negative outcomes in women, including body dissatisfaction, shame and reduced exercise adherence (Huellemann et al., 2021; Panão & Carraça, 2020). Therefore, the present study focused on women, for whom appearance motive carries a pronounced sociocultural burden.

According to the self‐determination theory (SDT) (Hagger & Chatzisarantis, 2008; Martela, 2020; Ryan & Deci, 2000), exercising for appearance is driven by controlled (e.g. extrinsic) motivation, where individuals feel compelled to exercise rather than genuinely wanting to (Cox et al., 2019; Teixeira et al., 2012; Vartanian et al., 2012). In line with this, more recent studies showed how guilt and shame may stimulate appearance‐based exercise, especially in women (Czepczor‐Bernat, 2022; Gilchrist et al., 2018; Hurst et al., 2017; Lucibello et al., 2020). In contrast, exercising for functional reasons (i.e. health/fitness and/or enjoyment) is considered more autonomous, as it stems from personal values and intrinsic pleasure (Ingledew et al., 2014; Panão & Carraça, 2020). Autonomous motivation supports long‐term adherence and positive psychological outcomes, while controlled motivation tends to undermine them (Brunet & Sabiston, 2011; Huellemann et al., 2021; Ingledew et al., 2014; Janssen et al., 2019; Panão & Carraça, 2020). Furthermore, this autonomous‐to‐controlled continuum is sustained by the satisfaction of three basic psychological needs: autonomy (a sense of volitional choice), competence (feeling effective in one's actions) and relatedness (feeling connected to others; Ryan & Deci, 2000; Teixeira et al., 2012). Health/fitness motives occupy an intermediate position along this continuum, that is, when individuals value their health and connect exercise to personal well‐being, exercise takes autonomous form (Teixeira et al., 2012). However, when exercise is experienced as an externally imposed obligation (e.g. GP prescribing physical activity) or guilt, it may reflect introjected or external regulation (Ingledew & Markland, 2008).

### Mood, motives and exercise engagement (H2)

Notably, the relationship between exercise and mood is bidirectional (Bourke et al., 2021; Chan et al., 2019; Childs & de Wit, 2014; Hearing et al., 2016; Tang et al., 2024). Importantly, though, mood states do not translate automatically into exercise behaviour, and affective responses to it vary across individuals (Chan et al., 2019; Craft et al., 2014). On the *exercise* → *mood* link, people exercising for functional reasons (health, enjoyment) report more positive affect following a session compared to those exercising for appearance (Englert & Rummel, 2016; O'Hara et al., 2014; Raedeke et al., 2007). In contrast, appearance‐related concerns during exercise tend to overshadow the positive emotions connected to it (Homan & Tylka, 2014). On the *mood* → *exercise* link, whether positive mood state is actually linked to subsequent exercise behaviour may strongly depend on the motive to exercise with functional motives expected to strengthen this link compared to appearance motives (Kwan & Bryan, 2010; Rhodes & Kates, 2015; Stevens et al., 2020).

The selection of exercise motives and mood as the two main constructs of this study is grounded in both theoretical and empirical considerations. From a theoretical point, SDT posits that the quality of motivation, not merely its quantity, shapes the affective outcomes of behaviour (Teixeira et al., 2012). Mood, in turn, is not only a potential outcome of exercise but also a key mechanism sustaining or undermining behavioural engagement over time, as affective responses to exercise predict future exercise intentions and long‐term adherence (Kwan & Bryan, 2010; Rhodes & Kates, 2015; Stevens et al., 2020). Critically, the interplay between these two constructs remains understudied in daily‐life contexts: while cross‐sectional and experimental studies suggest that motives moderate the mood‐enhancing effects of exercise (Gaitan‐Sierra & Hyland, 2014; Raedeke et al., 2007), little is known about whether and how this dynamic unfolds in people's daily life.

### Capturing daily‐life dynamics: Ecological momentary assessment and network analysis

Most previous research relied on retrospective or cross‐sectional studies (Bourke et al., 2023; Daley & Huffen, 2003; Kanning & Schlicht, 2010; Kanning & Schoebi, 2016; Liao et al., 2017; Schultchen et al., 2019; Smith et al., 2020); thus, the present study tracks in‐the‐moment mood and exercise engagement using smartphone‐based Ecological Momentary Assessment (EMA). Participants are prompted with a set of questions every day, multiple times a day (LePage & Crowther, 2010; MacIntyre et al., 2020; Shiffman et al., 2008; Smith & Juarascio, 2019). Compared to laboratory settings or diaries, EMA offers greater ecological validity and reduces memory bias. For these reasons, smartphone EMA is increasingly used to study everyday behaviours such as eating, snacking (Barchitta et al., 2022; Mason et al., 2020; Maugeri & Barchitta, 2019; Pimpini et al., 2023) and exercise (Comulada et al., 2018; Dunton et al., 2018; Kanning & Schlicht, 2010). Crucially, time series that EMA yields are not only capturing behaviour in real time but also allow to investigate temporal relationships within and between variables. Multilevel vector autoregressive (mlVAR) approach (Epskamp et al., 2018) is used to model how each variable predicts itself and others at the same assessment (contemporaneous) and from one assessment to the next (temporal, *t* − 1 → *t*). Applied to mood and exercise, the network approach moves beyond simply establishing a link between the two variables.

### The present study

Taken together, this study aims to explore the interplay between exercise motives (trait), exercise engagement (state) and mood (state) in daily life. The hypothesis is tested that functional motives (i.e. health/fitness and enjoyment), compared to appearance motives, predict greater exercise engagement (frequency, intensity and duration). As motives are trait assessed once at baseline, H1 concerns a between‐person (Level‐2) effect. Additionally, exercise motives are hypothesized to moderate the link between mood and exercise engagement (H2; cross‐level interaction). Finally, multilevel networks examine the within‐person dynamics between mood and exercise distinguishing same assessment (contemporaneous network) from prospective (temporal network) assessment.

## METHOD

### Procedure

The present study was composed of three phases (see Figure 1): In phase one, after giving informed consent, participants' demographics and inclusion criteria were assessed via an online questionnaire (Qualtrics). In phase two, eligible participants completed the Reasons for Exercise Inventory (REI; Silberstein et al., 1988) and the International Physical Activity Questionnaire (IPAQ; Booth, 2000) online (Qualtrics). Afterwards, participants received an instruction guide for installing and using the EMA application on their smartphone (Avicenna Research, https://avicennaresearch.com/). The final phase of the study consisted of a 2‐week EMA protocol always starting on a Monday. *Each smartphone notification required about 2–3 min to complete and*, if participant did not respond within 15 min, two reminders were sent 15 min apart. In total, 45 min elapsed between the first notification and the response window following the second reminder. Every day, participants received eight equidistant smartphone notifications between 8 AM and 10 PM. More specifically, mood was assessed eight times per day (i.e. 8:00, 10:00, 12:00, 14:00, 16:00, 18:00, 20:00, 22:00), while physical activity was assessed three times a day (i.e. 12:00, 18:00, 22:00). Participants spent approximately 15–20 min per day using the smartphone app. To ensure an adequate level of compliance, and to check for potential technical issues with the app, participants were contacted via email twice a week. Upon study completion, participants were debriefed via email about the goals of the study and received compensation (€20 voucher or university research credits). Notably, the present dataset was collected as part of a larger EMA study assessing people's lifestyle. That is—next to exercise and mood—eating, drinking, smoking and social behaviours were assessed daily.

**FIGURE 1:**
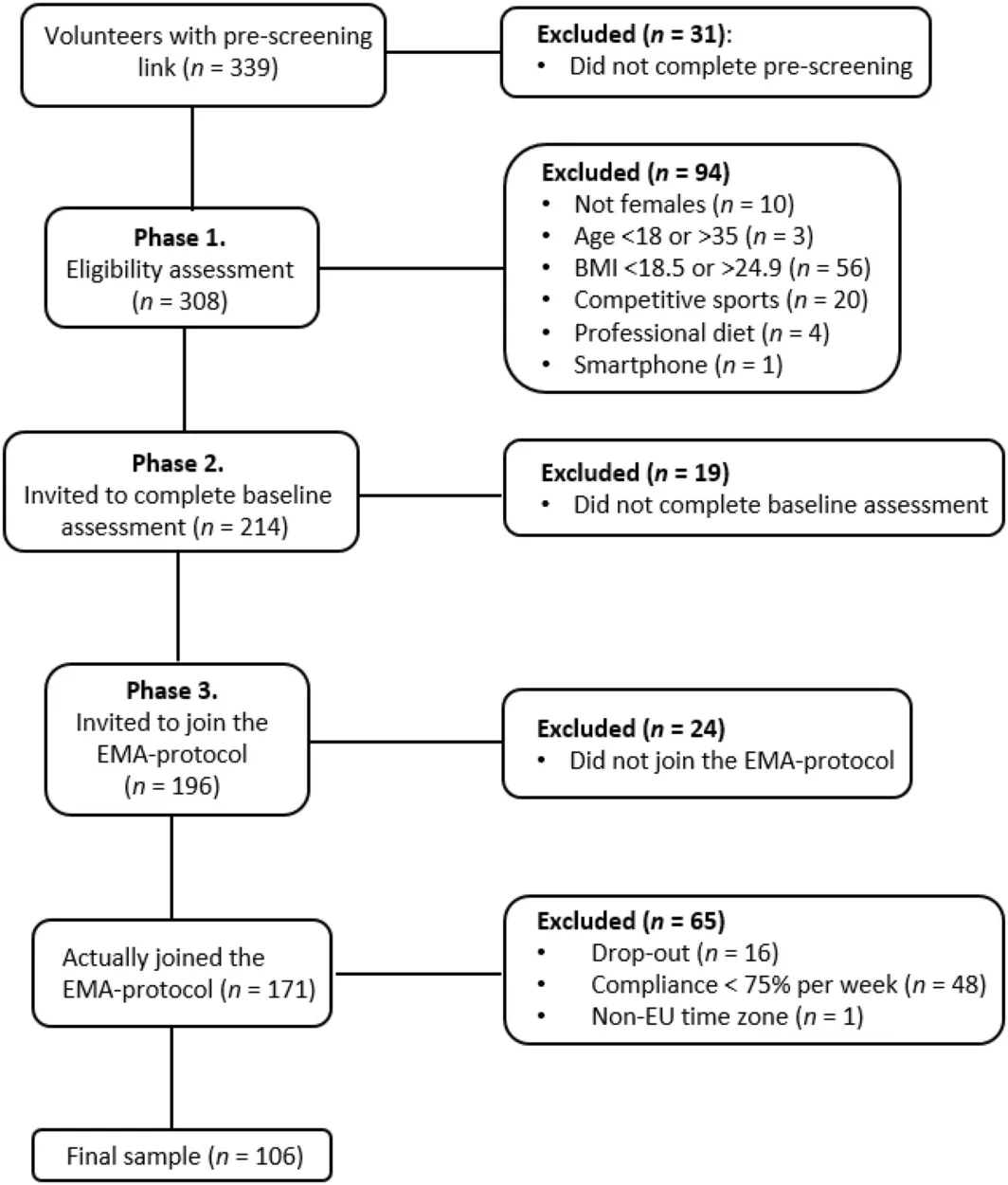
Participant selection. One participant was excluded from the analyses as they repeated technical issues uploading the data.

### Participants

In total, 339 individuals volunteered for participation. Of these, 214 met the inclusion criteria: female (sex assigned at birth), 18–35 years old, healthy body mass index [BMI]: 18.5–24.9, not on a professional diet, not pregnant, not practicing sport at a competitive level. Participants were recruited through paper flyers distributed around campus and posts on social media (e.g. Instagram, LinkedIn). Participants were recruited in the Netherlands. To ensure enough data per participant, a minimum compliance rate of 75% was set for each measurement week. The final sample consisted of 106 participants, which is in line with a priori, simulation‐based power analysis. As power in EMA designs depends primarily on the number of Level‐1 observations rather than the participant count, the simulation was calibrated to the present study: 106 participants each contributing up to 42 exercise prompts (3/day over 14 days), yielding 4452 Level‐1 observations, with variances set to reproduce the observed intraclass correlation (ICC = .10). Effect sizes were kept small (*β* = .02–.20), as is typical for within‐person mood–behaviour associations in EMA. With 1000 iterations at *α* = .05, the design had ≥80% power to detect a mood × motive cross‐level interaction at *β* = .06.

The study was preregistered through AsPredicted (https://aspredicted.org/gz5c‐m8nv.pdf). See Figure 1 for participant selection and Table 1 for participant characteristics.

**TABLE 1:** Participant characteristics.

Variable	*M*	SD	Range (min, max)	%
Age	24.51	3.44	19–35	
BMI	21.72	1.82	18.59–24.90	
Appearance motive (REI)	4.16	1.30	1.13–6.88	27
Health/fitness motive (REI)	5.14	.89	1–6.75	35
Enjoyment motive (REI)	4.24	.97	1.29–6.14	38
Low physical activity (<600 MET; IPAQ)	353.85	151.17	155.50–506.50	7
Moderate physical activity (600–3000 MET; IPAQ)	1794.82	649.12	642–2923.50	55
Vigorous physical activity (>3000 MET; IPAQ)	5332.52	2135.44	3106.50–11,466	38
European				83
Non‐European				17

*Note*: REI scores are measured using a scale from 1 to 7 (1 = Not important at all; 7 = Extremely important), while IPAQ scores are measured in MET minutes/week. MET represents the amount of energy expended carrying out physical activity.

Abbreviations: BMI, body mass index; IPAQ, International Physical Activity Questionnaire; MET, Metabolic Equivalent of Task; REI, Reasons for Exercise Inventory (Silberstein et al., 1988).

### Baseline measurements

#### International physical activity questionnaire

To obtain an indication of the physical activity level in this sample, a modified version of the short IPAQ (Booth, 2000) was used. This is a seven‐item measure that gathers self‐reported information on the time spent in vigorous PA, moderate PA, walking and sitting in a typical week (MacIntyre et al., 2020). Notably, IPAQ scores are measured in Metabolic Equivalent of Task (MET) reported in minutes per week. MET represents the amount of energy expended carrying out a specific physical activity. Each type of physical activity corresponds to a specific value, and the final score is obtained by multiplying MET minutes/week by the number of minutes per day and the number of days per week spent on that activity. The validity of the IPAQ (*α =* .80) has previously been established (Craig et al., 2003; Meh et al., 2021) against objectively measured PA (Ball et al., 2014; MacIntyre et al., 2020). For this study, the IPAQ was administered solely to characterize the sample.

#### Reasons for exercise inventory

Participants' motives to exercise were assessed by the REI (Silberstein et al., 1988), consisting of 24 items reflecting seven different motives: weight control, attractiveness, body tone, health, fitness, enjoyment and improved mood. Participants rated these motives on perceived importance on a 7‐point Likert scale ranging from 1 (= *Not at all important*) to 7 (= *Extremely important*) (Vartanian et al., 2012). Higher scores on each subscale indicated higher levels of the respective motive (Trott et al., 2020). Furthermore, the seven subscales can be collapsed into three distinct categories, resulting in one overall index of appearance‐related exercise motive and two functionality‐related indexes of exercise motive (i.e. health/fitness and enjoyment; O'Hara et al., 2014). This categorization (REI) showed rather high internal consistency for appearance‐related reasons (*α =* .85), health/ fitness (*α =* .85) and enjoyment (*α* = .76) (Prichard & Tiggemann, 2008). Notably, item 16 of the appearance subscale (i.e. ‘to maintain my appearance’) was excluded from the analyses due to incorrect phrasing (‘to maintain my well‐being’). Cronbach alpha in the current sample was .73. Finally, to identify each participant's *predominant* exercise motive, the highest score was selected.

#### EMA measurement

##### Exercise

Participants received three notifications per day every day: (a) at 12.00 (*Have you exercised this morning, between 6.00 and 12.00?*) (b) at 18.00 (*Have you exercised this afternoon, between 12.00 and 18.00?*) and (c) at 22.00 (*Have you exercised this evening, between 18.00 and 22.00?*). If the answer was ‘yes’, two follow‐up questions appeared on screen about exercise intensity (0 = No effort; 100 = Vigorous effort) and exercise duration (0 = 10 min; 100 = >90 min).

##### Mood

To investigate participants' mood (How do you feel at this moment?), nine mood states were presented. More specifically, two positive mood states (joyful/happy, calm/relaxed) and seven negative mood states (angry/annoyed, tired/fatigued, tense/stressed, bored, guilty/ashamed, anxious/scared, sad/depressed). Participants could select at least one and up to four mood states and, for each selected state, indicate the intensity using a 100‐mm VAS ranging from 0 (not at all) to 100 (very much). This assessment was chosen to maximize sensitivity to within‐day mood variability. That is, by letting participants select their most appropriate state‐specific mood, it reduces measurement reactivity and floor effect (Shiffman et al., 2008).

### Psychometric properties of EMA items

McDonald's omega (*ω*) was estimated separately for within‐ and between‐subject variance to assess the internal consistency of the four EMA items, that is, overall mood, exercise frequency, exercise intensity, exercise duration. Unlike Cronbach's alpha, *ω* does not assume tau‐equivalence and accommodates the hierarchical structure of EMA data by computing reliability at each level independently. Internal consistency was acceptable to good at both levels (*ω*_within = .87; *ω*_between = .86). Factor loadings were strong for the three exercise engagement items (*λ*_within = .92–.96; *λ*_between = .90–.94), whereas overall mood loaded more weakly on the common factor (*λ*_within = .16; *λ*_between = .19), reflecting distinction between mood and exercise engagement as related but non‐redundant constructs.

### Data analysis

All analyses were conducted using RStudio 2024.12.1.

Notably, as mood and physical activity (intensity and duration) were not sampled equally—eight and three times per day, respectively—mood scores were aggregated into the three PA time points: 12:00, 18:00, 22:00.

### Overall mood

To compute the *overall* mood score, all selected positive mood scores and all selected negative mood scores were averaged. All negative mood states were assigned a negative sign. Then, we calculated a net overall mood score using this formula:

$$\mathrm{Net}\;\text{Score}=\left(\text{Average Positive Mood}\right)-|\text{Average Negative Mood}|.$$

Finally, overall mood score ranges from −100 to 100.

#### Between‐subject cross‐level analysis

##### *H1*: Functional motives—compared to appearance motives—predict greater exercise engagement

EMA data on exercise were aggregated to obtain summary scores for each participant. Aggregates for exercise frequency reflected how often (times per day) each participant engaged in physical exercise throughout the study (14 days). Aggregates for intensity and duration represented the average exercise intensity and duration per exercise session per participant. Three separate linear regression analyses were performed using exercise engagement (frequency, intensity, duration) as dependent variables, and exercise motives (enjoyment, health/fitness, and appearance) as predictors.

##### *H2*: Higher (more positive) mood, in interaction with motives to exercise, predicts greater exercise engagement

Three analyses (i.e. one per exercise engagement variable: frequency, intensity and duration) were conducted using one of the exercise engagement variables (i.e. frequency, intensity, duration) as the dependent variable, with overall mood (person‐mean centred) as the Level‐1 predictor, and exercise motives (i.e. health/fitness, enjoyment, appearance; grand‐mean centred) as Level‐2 predictors and moderators. Cross‐level interaction terms between mood and each motive were included to test whether motives moderate the within‐person mood–exercise association. For each analysis, four models were developed sequentially and evaluated against each other using Akaike's information criterion (AIC), where a lower AIC value signified a better fit for the updated model.

The data had a two‐level nested structure in which repeated EMA assessments (Level 1) were nested within participants (Level 2). The models were estimated on the raw, beep‐level (i.e. moment) EMA observations rather than on person‐ or day‐level: each row in the analysis corresponded to a single exercise prompt, and the random intercept for participant captured the resulting dependency among observations from the same person. For exercise frequency, all logged exercise prompts were modelled (*N* = 3399 observations from 98 participants), whereas the intensity and duration models were restricted to those prompts at which an exercise session was reported and these follow‐up items were completed (*n* = 729 exercise sessions). Because the three exercise motives were assessed once (baseline), they were strictly between‐person (Level‐2) variables with no within‐person variance, whereas overall mood varied both within and between persons and was therefore person‐mean centred to isolate its within‐person (Level‐1) component. Motives were grand‐mean centred as well. Predictors and outcomes were modelled *concurrently*, that is, mood and exercise engagement were drawn from the same assessment occasion (time *t*), so that the MLM tested the same‐moment association between mood and exercise engagement and its moderation by motive.

###### Model 0

First, a null—unconditional—model was built, which only contained the exercise engagement variable (frequency, intensity or duration) as the dependent variable and a random intercept to account for between‐subjects variability. No predictors were added at this stage. This model partitioned the total variance into a within‐person (Level‐1; moment) and a between‐person (Level‐2) component and the resulting intraclass correlation (ICC) indexed the proportion of variance attributable to stable between‐person differences.

###### Model 1

Next, the main predictor variable (overall mood, person‐mean centred)—fixed effect—was added. Model 1 also included a random intercept.

###### Model 2

It was tested whether slopes should remain fixed or vary randomly to check whether the within‐person effect of mood on exercise engagement differed across participants.

###### Model 3

The remaining between‐subjects predictors of interest (i.e. the three exercise motives)—as well as cross‐level interaction terms—were added to test main effects and interaction effects.

#### Within‐subject momentary network analysis

Network analysis was conducted, in addition to preregistered analysis, to assess momentary within‐person associations between exercise and mood using time‐series smartphone EMA data. That is, examining not only momentary between variables dynamics (same assessment) but also temporal (*t* − 1 → *t*) between variable dynamics. For this analysis, only the EMA data, not the baseline scores, was used.

##### Network analysis: Assumptions

Prior to estimate the networks (Borsboom et al., 2021; Epskamp et al., 2018), four assumptions were checked: stationarity, variability, multivariate normality and equidistant assessment times. Stationarity assumption states that time series holds stable means over time, that is, no upward or downward trend should be observed. In this study, we checked whether means changes (i.e. the presence of linear trends) by regressing each variable onto a time variable where each level represents an assessment moment for each participant separately. If time was a significant predictor (alpha = .05) for a particular variable for a particular participant that variable was considered non‐stationary for that participant, and the residuals of the regression model replaced the original variable. As for variability, the model assumes some variability for each variable over time. Therefore, entire participants and/or variables containing zero variance were excluded from the analysis. Furthermore, to check for multivariate normality of each variable, the Kolmogorov–Smirnov test was used (Goodman, 1954).

Finally, the mlVAR model also assumes equidistant assessment times; this assumption is intrinsically violated due to the night interval between the last prompt of day one and the first prompt of the following. To overcome this, relationships between the last prompt of day one and the first of the following were not estimated.

##### Network analysis: Estimation

To estimate networks, the *mlVAR* function of the R package *mlVAR* (Epskamp et al., 2018) was used. The mlVAR model is the multi‐level extension of the vector autoregressive model (VAR) where each variable at time *t* − 1 is regressed on itself and to all other variables at time *t*. All relations are corrected for the influence of all other variables. This model comprises three networks: temporal, contemporaneous and between‐subject network (not estimate in this study). The temporal network shows the temporal relations between the variables (from *t* − 1 to *t*). With mlVAR models, the nested structure of the data can be used to estimate idiographic deviations from the sample's coefficients (i.e. random effects). Residuals of the temporal network were used to build the contemporaneous network consisting of partial correlations showing the relations between variables within the same time point. Linear mixed‐effects regression model (lmer) was used to estimate multilevel relationships. In both networks—temporal and contemporaneous—alpha was set at .05 and Bonferroni corrected to adjust for multiple testing of the null hypothesis (i.e. a relationship exists between two variables).

## RESULTS

### Data cleaning

Notably, eight participants were excluded from the analyses because they reported no exercise engagement throughout the EMA protocol. Finally, one participant reported extremely low scores on the enjoyment and health/fitness REI subscales. These scores were Winsorized (Blaine, 2018; Kwak & Kim, 2017), that is, the two scores below the fifth percentile were replaced with the value of the fifth percentile. Finally, 98 participants were included in the analysis.

### Compliance rate

Overall, average compliance to the EMA protocol was high (90.46%, SD = 6.43, Min = 75.89, Max = 100). Moreover, compliance did not differ significantly between Week 1 (90.68%) and Week 2 (90.24%), *t* (96) = 1.05, *p* = .362.

### EMA data

In total, 3399 observations of different exercise episodes were collected from 98 participants. Across participants, exercise engagement throughout the 14‐day EMA‐protocol was moderately high, with a mean frequency of 8.7 sessions (SD = 6), intensity of 55.08 (SD = 19) and duration of 49 min (SD = 18). Notably, these were first averaged per participant (across all time points) and then across participants. Exercise intensity and duration showed a moderate correlation (*r* = .50, *p* < .01), while the correlations between exercise frequency and duration (*r* = .16, *p* = .10), and exercise frequency and intensity (*r* = .14, *p* = .15) were not statistically significant. Finally, overall mood was, on average, low (*M* = 8.03, SD = 6.05), reflecting an equal distribution between positive (*M* = 69.14, SD = 6.24) and negative mood scores (*M* = 58.59, SD = 5.87).

### *H1* : Functional motives—compared to appearance motives—predict greater exercise engagement

Three multiple linear regression analyses were conducted to examine whether appearance, health/fitness and enjoyment motives predicted exercise frequency, intensity and duration. For exercise frequency, the overall regression model was significant, *F* (3, 94) = 3.69, *p* = .015, explaining 10.5% of the variance (*R* ^2^ = .11, adjusted *R* ^2^ = .08). Enjoyment motive was the only significant predictor of exercise frequency (*B* = 1.96, SE = .75, *t* = 2.61, *p* = .011), whereas appearance motive (*p* = .646) and health/fitness motive (*p* = .816) were not significantly associated with exercise frequency. The regression model predicting exercise intensity was not significant, *F* (3, 94) = .82, *p* = .488 (*R* ^2^ = .03, adjusted *R* ^2^ = −.01). None of the exercise motives significantly predicted exercise intensity (all *p*s > .20). For exercise duration, the overall regression model was significant, *F* (3, 94) = 5.50, *p* = .002, explaining 14.9% of the variance (*R* ^2^ = .15, adjusted *R* ^2^ = .12). Enjoyment motive positively predicted exercise duration (*B* = 8.83, SE = 2.24, *t* = 3.94, *p* < .001), whereas health/fitness motive was negatively associated with exercise duration (*B* = −6.43, SE = 2.70, *t* = −2.38, *p* = .019). Appearance motive was not significantly associated with exercise duration (*p* = .235).

Overall, these findings partially supported H1. Consistent with our hypothesis, the enjoyment motive was associated with greater exercise frequency and longer exercise duration. Contrary to expectations, the health/fitness motive was negatively associated with exercise duration, while the appearance motive was unrelated to all three indicators of exercise engagement (Table 2).

**TABLE 2:** Overview of the results for H1.

Variable	*B* (SE)	*β*	*t*	*p*	95% CIs
Exercise frequency					
Appearance motive	−.20 (.45)	−.04	−.46	.646	−1.10, .69
Health/fitness motive	.21 (.90)	.02	.23	.815	−1.58, 2.01
Enjoyment motive	1.95 (.75)	.30	2.60	.010*	.466, 3.44
Exercise intensity					
Appearance motive	−1.20 (1.49)	−.08	−.80	.421	−4.16, 1.75
Health/fitness motive	−2.01 (2.98)	−.08	−.67	.501	−7.94, 3.91
Enjoyment motive	3.16 (2.47)	.155	1.28	.203	−1.74, 8.80
Exercise duration					
Appearance motive	1.61 (1.35)	.11	1.19	.234	−1.06, 4.29
Health/fitness motive	−6.43 (2.70)	−.26	−2.38	.019*	−11.79, −1.06
Enjoyment motive	8.83 (2.39)	.44	3.94	<.001**	4.38, 13.27

*Note*: *B*, unstandardized coefficients; *β*, standardized coefficients.

* *p* < .05.

** *p* < .001.

### *H2* : Higher (more positive) mood, in interaction with motives to exercise, predicts greater exercise engagement

Multilevel models were estimated separately for each exercise engagement outcome (frequency, intensity, duration), with person‐mean‐centred mood as the Level‐1 predictor and grand‐mean‐centred exercise motives as Level‐2 moderators. Models were fitted to moment‐level observations (assessments nested within persons), relating mood to exercise engagement concurrently. Null models supported multilevel modelling (ICC = .10 frequency, .43 intensity, .37 duration). For frequency (*N* = 3399), a likelihood‐ratio test favoured a random slope for mood (*p* = .016), but the random‐slope model failed to converge and was near‐unidentifiable—due to a scale mismatch between the binary outcome and continuous predictor—so a random‐intercept‐only model was retained. For intensity and duration (*N* = 729), random‐slope models likewise failed to converge and did not improve AIC; random‐intercept‐only structures were retained throughout (see Table 3).

**TABLE 3:** Overview of the results for H2.

Predictor	*B* (SE)	*β*	*t*	*p*	95% CI
Exercise frequency (PA)					
Overall mood	.0003 (.0001)	.04	1.90	.061	−.000, .001
Appearance motive	−.008 (.011)	−.03	−.71	.479	−.030, .014
Health/fitness motive	−.002 (.021)	−.00	−.10	.920	−.044, .040
Enjoyment motive	.017 (.019)	.04	.93	.353	−.019, .054
Mood × appearance	.0001 (.0001)	.02	1.14	.260	−.000, .000
Mood × health/fitness	−.0002 (.0002)	−.02	−.92	.361	−.001, .000
Mood × enjoyment	.0001 (.0002)	.01	.48	.635	−.000, .000
Exercise intensity					
Overall mood	−.001 (.012)	−.01	−.06	.950	−.025, .024
Appearance motive	−1.056 (1.407)	−.05	−.75	.455	−3.81, 1.70
Health/fitness motive	−.161 (2.752)	−.01	−.06	.953	−5.56, 5.23
Enjoyment motive	1.261 (2.391)	.05	.53	.599	−3.43, 5.95
Mood × appearance	.022 (.010)	.07	2.14	.033*	.002, .041
Mood × health/fitness	−.015 (.019)	−.03	−.80	.426	−.053, .022
Mood × enjoyment	.008 (.016)	.02	.48	.632	−.024, .039
Exercise duration					
Overall mood	.013 (.013)	.03	1.01	.315	−.012, .039
Appearance motive	2.144 (1.210)	.12	1.77	.080	−.23, 4.52
Health/fitness motive	−4.683 (2.377)	−.15	−1.97	.052	−9.34, .025
Enjoyment motive	7.773 (2.059)	.30	3.78	<.001**	3.74, 11.81
Mood × appearance	.005 (.011)	.02	.45	.655	−.016, .026
Mood × health/fitness	−.019 (.020)	−.04	−.96	.337	−.059, .020
Mood × enjoyment	.009 (.017)	.02	.51	.612	−.025, .042

*Note*: *B*, unstandardized coefficients; *β*, standardized coefficients.

* *p* < .05.

** *p* < .001.

Contrary to H2, within‐person mood did not predict exercise frequency (*B* = .0003, *β* = .04, *t* = 1.90, *p* = .061), and no mood × motive interaction was significant (all *p*s > .25). For exercise intensity, mood showed no main effect (*B* = −.001, *p* = .950), but the appearance motive moderated the mood–intensity association (*B* = .022, SE = .010, *β* = .07, *t* = 2.14, *p* = .033; 95% CI [.002, .041]). That is, participants higher in appearance motive showed a stronger positive link between mood and exercise intensity (see Figure 2). No other intensity interactions were significant (all *p*s > .40). For exercise duration, neither mood (*B* = .013, *p* = .315) nor any mood × motive interaction was significant (all *p*s > .33). However, the enjoyment motive predicted longer exercise duration as a between‐person main effect (*B* = 7.77, SE = 2.06, *β* = .30, *t* = 3.78, *p* < .001; 95% CI [3.74, 11.81]), replicating the H1 between‐person effect within the EMA data.

**FIGURE 2:**
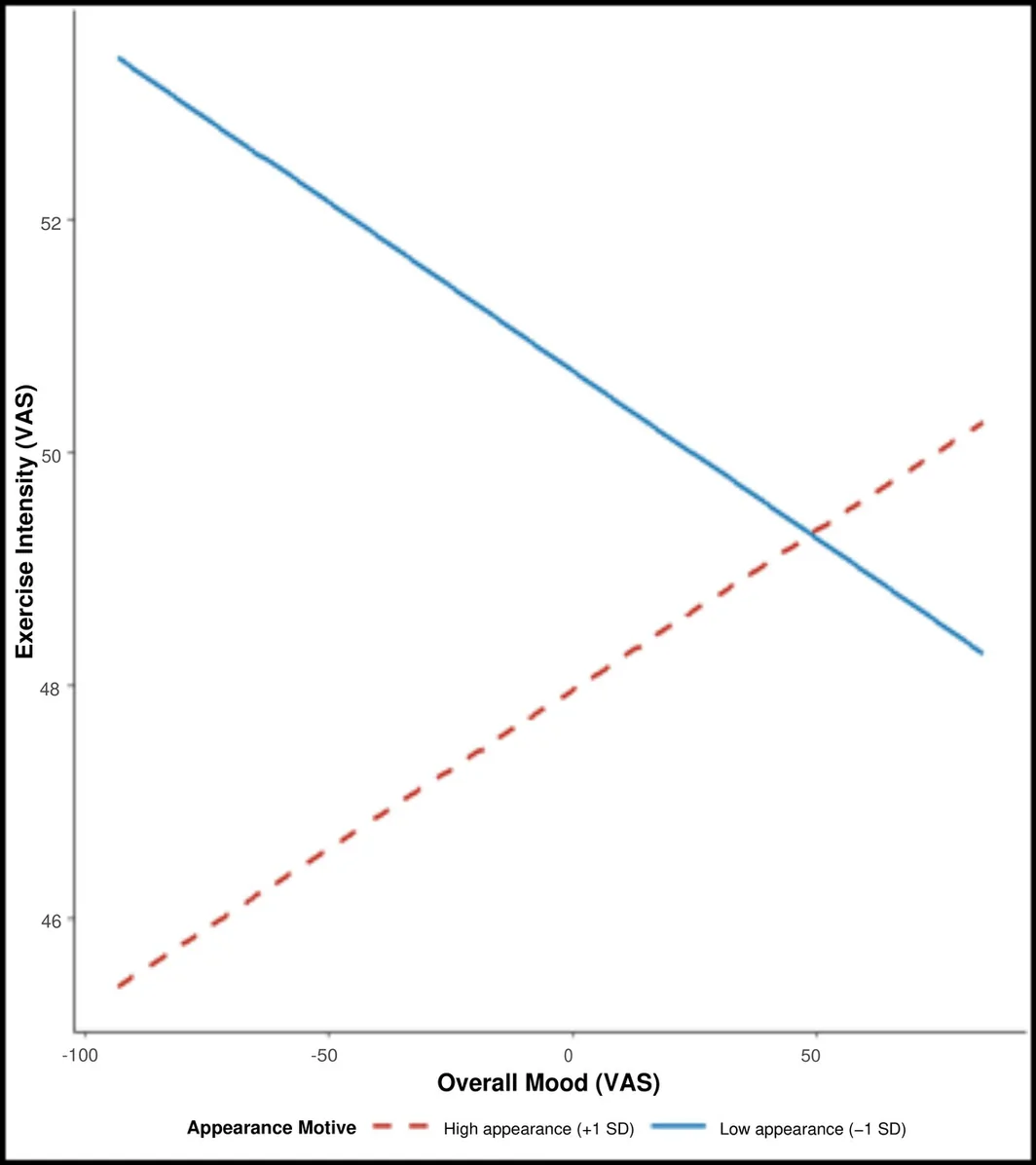
Within‐person relationship between overall mood and exercise intensity, moderated by the appearance motive.

### Network analysis results

#### Assumptions check

In total, 98 participants and 3 variables per participant were analysed, resulting in 294 time series. No participant, or single variable, was excluded due to stationarity (i.e. linear upward or downward trend over time). Instead, 65 participants (66.3%) had to be excluded due to lack of variability in two out of three variables: exercise duration and intensity. In other words, networks could be estimated for 33 participants (33.6%) only. Finally, normality assumption was not violated considering the final subsample.

#### Networks estimation

The contemporaneous network indicated a strong positive relation (*ω* = .89) between exercise duration and intensity. In other words, within the same assessment, participants who exercised more intensely tended to exercise for longer periods. No other relation involving overall mood reached significance.

In contrast, the temporal network showed that more positive mood at prior assessment predicted slightly greater exercise intensity (*β* = .09) and duration (*β* = .1) in the following assessment. Moreover, more positive mood predicted itself moving from previous to following assessment (*β* = .13). All these effects were rather small. See Figure 3 for an overview.

**FIGURE 3:**
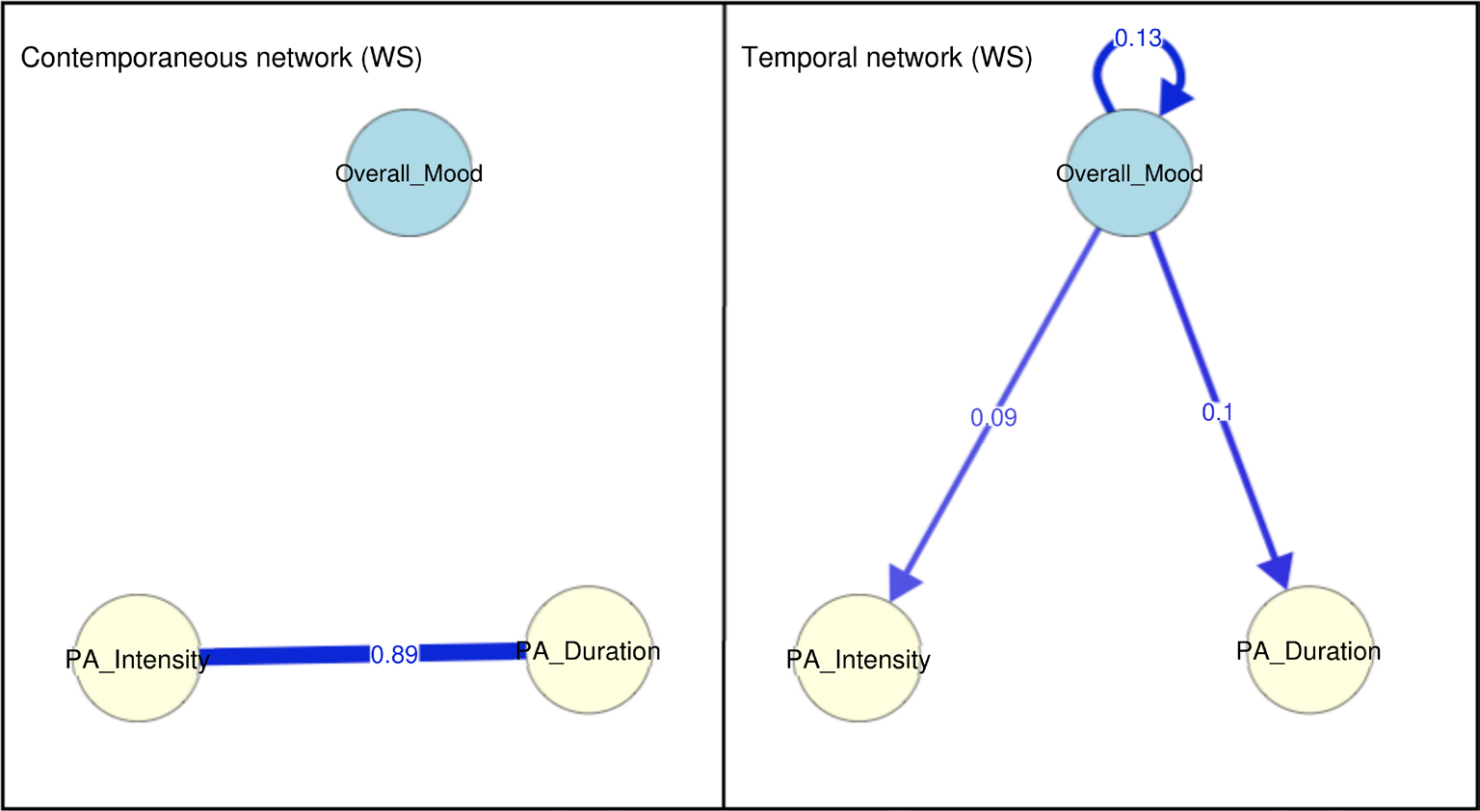
Graphical representation of the contemporaneous and temporal networks illustrating the relationship between overall mood, PA duration and PA intensity. Notably, 33 participants of 98 were included in this analysis. The rest reported insufficient PA.

## DISCUSSION

The goal of the present study was to examine the link between exercise motives, exercise engagement and mood among women with a healthy weight. It was hypothesized that (H1) functional motives, compared to appearance motives, predict greater exercise engagement. Moreover, it was hypothesized that (H2) higher (i.e. more positive) mood, in interaction with motives to exercise, predicts greater exercise engagement. Additional network analysis investigates the within‐subject relationship between mood and exercise both within the same assessment and from the current to the next assessment.

As a result, in line with H1, functional motives, especially enjoyment, were linked to higher exercise engagement, with the enjoyment motive positively linked to exercise frequency and duration. Moreover, in contrast to H1, the health/fitness motive correlated negatively with exercise duration.

As for H2, the hypothesis was not supported: contrary to expectation, functional motives did not strengthen the association between mood and exercise engagement. Instead, the only significant moderation involved the appearance motive, which moderated the mood–exercise intensity relationship. That is, participants scoring high on the appearance motive reported a stronger positive link between mood and exercise intensity. Mood itself did not predict exercise engagement at momentary level. Lastly, the enjoyment motive positively predicted exercise duration as a between‐person effect, with participants more strongly motivated by enjoyment exercising for longer. Notably, this effect replicates H1 rather than reflecting the mood‐dependent pattern predicted under H2.

The finding that enjoyment motives predict exercise frequency aligns well with the SDT (Hagger & Chatzisarantis, 2008), which posits that intrinsic motives—specifically enjoyment—significantly influence people's behaviour and overall well‐being (Brunet & Sabiston, 2011; Ingledew et al., 2014; Teixeira et al., 2012). This finding is further supported by existing literature on the so‐called hedonic theory linking the feeling of pleasure experienced before, during and after an exercise session (Berger et al., 2023; Lee et al., 2015; Rhodes & Kates, 2015; Teixeira et al., 2021). People are more likely to repeat activities that elicit positive affect and avoid those associated with negative affect, a pattern that has been consistently linked to exercise adherence and exercise frequency (Hagberg et al., 2009; Jekauc, 2015; Schneider & Cooper, 2011; Schwaneberg et al., 2017; Woods et al., 2012). Moreover, studies have shown that individuals who find intrinsic enjoyment in their (physical) activities are more likely to maintain such a habit for a longer time, leading to better health outcomes and psychological well‐being (Fennell et al., 2022; Strasser & Fuchs, 2015). Thus, fostering intrinsic enjoyment in physical exercise is essential for encouraging regular and sustained activity.

Unexpectedly, health/fitness motives correlated negatively with exercise duration. These results, although the effect was small, showed that individuals who exercise for health/fitness reasons tend to exercise for shorter duration. These findings can also be explained by the dual process model (Houlihan, 2018; Phipps et al., 2021), that is, participants' conscious motive to exercise for health/fitness reasons were overridden by in‐the‐moment affective factors linked to lack of immediate reward. In line with this, positive health outcomes have been reported with ‘only’ 10–30 min of physical activity per day, or a few sessions per month (Herbert et al., 2020; Thijssen et al., 2022; Wilson et al., 2001). Finally, while appearance motives typically involve long, low‐intensity sessions for weight loss, health and fitness motives target performance gains favouring shorter—not necessarily more intense—workouts (Coates et al., 2023; Smith et al., 2016).

As for H2 findings, the predicted moderation was not observed for functional motives; instead, and contrary to the hypothesized direction, the only significant interaction involved the appearance motive. Specifically, women higher in appearance motive showed a stronger concurrent (same‐moment) within‐person association between their mood and how intensely they exercised. Because the multilevel models related mood and intensity at the same assessment, this finding describes a same‐moment coupling rather than a directional, mood‐enhancing effect of exercise; the temporal ordering of mood and exercise is addressed separately in the network analyses below. This concurrent pattern is consistent with objectification theory and with evidence that appearance‐driven exercisers monitor their bodies in affectively charged ways (Greenleaf, 2005). For these individuals, momentary mood and exercise intensity may be more tightly linked—whether because intense exercise accompanies appearance‐related affect or because mood and intensity co‐fluctuate for other reasons—than they are for women exercising for functional reasons.

Moreover, the finding that enjoyment motive predicted longer exercise duration as a between‐person effect replicates the H1 trait‐level relationship within the EMA data set rather than supporting H2. The result aligns closely with SDT (Deci & Ryan, 2008) under which enjoyment is the prototypical intrinsic motive and the form of motivation linked to sustained engagement. Intrinsic, enjoyment‐based motivation is more strongly associated with long‐term exercise adherence and maintenance than externally oriented motives such as appearance or weight control (Teixeira et al., 2012; Ingledew & Markland, 2008), and enjoyment specifically predicts adherence even when it is not the most highly endorsed reason for exercising (Rodrigues et al., 2020). That enjoyment predicts exercise duration rather than frequency or intensity fits the picture observed in H1. The robustness of this between‐person effect across both the cross‐sectional (H1) and intensive longitudinal (EMA) data strengthens confidence that enjoyment is a meaningful drive of exercise duration.

Network analyses (mlVAR) extended the multilevel findings by characterizing the within‐person temporal ordering of mood and exercise engagement that the concurrent models could not resolve. In the *contemporaneous* network, exercise intensity and duration were strongly coupled within the same assessment (*ω* = .89), indicating that more intense sessions were also longer, whereas overall mood was not contemporaneously related to either engagement variable. The *temporal* network was more informative for the mood–exercise question: more positive mood at one assessment predicted slightly greater exercise intensity (*β* = .09) and duration (*β* = .10) at the following assessment, and mood showed positive autoregression (*β* = .13), tending to carry over from one assessment to the next. Although small, this directional pattern is consistent with affective theories of physical activity, which hold that anticipated and current positive affect motivate subsequent engagement, and with prior EMA work reporting that pleasant mood states precede increases in activity in everyday life (Kanning & Schlicht, 2010; Liao et al., 2017). Notably, the temporal effect ran from mood to subsequent exercise rather than the reverse, the direction predicted under H2 but not detectable in the concurrent models—suggesting that mood may operate as an antecedent of how intensely and how long people exercise, rather than (or in addition to) an immediate by‐product of it. This prospective mood‐to‐exercise pathway should be interpreted cautiously (i.e. 33 of 98 participants).

Contrary to our hypothesis, none of the exercise motives significantly predicted exercise intensity. This finding suggests that while the reasons underlying exercise participation may influence whether individuals choose to exercise and for how long, they may be less relevant in determining how intensely they exercise during a given session. Exercise intensity is likely shaped by more immediate situational and physiological factors, such as current energy levels, fitness capacity, time constraints or the specific type of activity performed, which may outweigh the influence of relatively stable motivational dispositions. Although SDT proposes that autonomous forms of motivation promote greater engagement in health behaviours (Ryan & Deci, 2000; Teixeira et al., 2012), the present findings indicate that this association may primarily manifest in exercise participation and persistence rather than exercise intensity. This interpretation is consistent with previous research suggesting that intrinsic motives are more strongly associated with exercise adherence and frequency than with the intensity of individual exercise sessions (Ingledew et al., 2014; Teixeira et al., 2012). Future studies should examine whether contextual variables, such as exercise modality, perceived exertion or environmental constraints, moderate the relationship between exercise motives and exercise intensity in daily life.

A major strength of the present study is the use of a relatively intensive EMA protocol (i.e. 8 beeps/day 8 for 14 days), which enabled the investigation of exercise motives, exercise engagement, and mood as they naturally occurred in daily life. Compared to retrospective questionnaires and laboratory‐based designs, this approach provides greater ecological validity while reducing recall bias. Furthermore, by combining multilevel modelling with network analysis, the study captured both between‐person associations and within‐person temporal dynamics, offering a more comprehensive understanding of the interplay between exercise, motives and mood as they occur.

Despite its strengths, this study presents limitations. For instance, the relatively short duration of the EMA protocol could not capture long‐term interplays between exercise and mood. Moreover, exercise duration should have been measured by its actual duration rather than by VAS, and mood assessment was too fragmented. Future EMA studies could assess positive and negative mood separately, each rated for intensity via a follow‐up item, and resolve the imbalance between negative and positive mental states. Information about menstrual cycle was not collected which represent a limitation given its link with exercise behaviours (Srinivasa Gopalan et al., 2024), motives (Ball et al., 2014) and (post‐exercise) mood (Pierson et al., 2021; Prado et al., 2021; Poromaa & Gingnell, 2014). Finally, reliance on self‐reported measures only is susceptible to social desirability response style, potentially affecting results. To overcome this, future research could provide participants with wearable activity‐tracking devices (e.g. smartphones, smartwatches, rings) and use the data from these devices in addition to self‐reported measures.

Taken together, this study advances the field by moving the motive–exercise–mood question from the retrospective and cross‐sectional designs to the momentary (multilevel models) and the lagged levels (temporal network). Findings suggest that programmes for healthy‐weight women should foreground genuinely enjoyable activities rather than health or appearance framing, that the same EMA prompts could be repurposed as a just‐in‐time tool timing encouragement to moments of elevated mood, and that exercise prescription should be tailored to a person's predominant motive since the mood benefit of intense exercise was confined to women high in appearance motive. Future EMA studies could explore two promising directions: examining exercise motives not only as static traits but also as dynamic states and incorporating body image assessment to provide a more comprehensive characterization of the sample, particularly for individuals exercising for appearance motives. This insight could be highly valuable for predicting dropout rates from weight loss programmes.

## AUTHOR CONTRIBUTIONS

**Leonardo Pimpini:** Conceptualization; investigation; writing – original draft; methodology; validation; visualization; writing – review and editing; software; formal analysis; data curation; project administration. **Ella Ferrand:** Conceptualization; methodology; investigation; formal analysis; writing – review and editing. **Codrin Mironiuc:** Formal analysis; visualization. **Jan Schepers:** Formal analysis; writing – review and editing. **Anne Roefs:** Funding acquisition; supervision; conceptualization; writing – review and editing; writing – original draft.

## ETHICS STATEMENT

This study received approval from the ethical review board of the Faculty of Psychology and Neuroscience at Maastricht University, the Netherlands (ERCPN, OZL_250_42_03_2022).

## Data Availability

Raw data, preprocessed data and analysis scripts are openly available: https://osf.io/pzjge/overview.
